# Probiotic *Lactobacillus rhamnosus* GG improves insulin sensitivity and offspring survival via modulation of gut microbiota and serum metabolite in a sow model

**DOI:** 10.1186/s40104-024-01046-z

**Published:** 2024-07-02

**Authors:** Tianle Gao, Ran Li, Liang Hu, Quanfang Hu, Hongmei Wen, Rui Zhou, Peiqiang Yuan, Xiaoling Zhang, Lingjie Huang, Yong Zhuo, Shengyu Xu, Yan Lin, Bin Feng, Lianqiang Che, De Wu, Zhengfeng Fang

**Affiliations:** 1 Key Laboratory for Animal Disease-Resistance Nutrition of China Ministry of Education, Animal Nutrition Institute, Sichuan Agricultural University, 211 Huimin Road, Wenjiang District, Chengdu, 611130 China; 2 Key Laboratory of Agricultural Product Processing and Nutrition Health (Co-construction by Ministry and Province), Ministry of Agriculture and Rural Affairs, College of Food Science, Sichuan Agricultural University, Ya’ an, 625014 China

**Keywords:** Gut microbiota, Insulin resistance, *Lactobacillus rhamnosus* GG, Lactation performance, Piglet, Sow

## Abstract

**Background:**

Sows commonly experience insulin resistance in late gestation and lactation, causing lower feed intake and milk production, which can lead to higher mortality rates in newborn piglets. The probiotic *Lactobacillus rhamnosus* GG (LGG) is known to improve insulin resistance. However, whether supplementing LGG can improve insulin sensitivity in sows and enhance lactation performance, particularly the early survival of offspring remains unclear. Hence, we explored the effects and mechanisms of supplementing LGG during late gestation and lactation on sow insulin sensitivity, lactation performance, and offspring survival. In total, 20 sows were randomly allocated to an LGG (*n* = 10) and control group (*n* = 10).

**Results:**

In sows, LGG supplementation significantly improved insulin sensitivity during late gestation and lactation, increased feed intake, milk production and colostrum lactose levels in early lactation, and enhanced newborn piglet survival. Moreover, LGG treatment significantly reshaped the gut microbiota in sows, notably increasing microbiota diversity and enriching the relative abundance of insulin sensitivity-associated probiotics such as *Lactobacillus*, *Bifidobacterium*, and *Bacteroides*. Serum metabolite and amino acid profiling in late-gestation sows also revealed decreased branched-chain amino acid and kynurenine serum levels following LGG supplementation. Further analyses highlighted a correlation between mitigated insulin resistance in late pregnancy and lactation by LGG and gut microbiota reshaping and changes in serum amino acid metabolism. Furthermore, maternal LGG enhanced immunity in newborn piglets, reduced inflammation, and facilitated the establishment of a gut microbiota.

**Conclusions:**

We provide the first evidence that LGG mitigates insulin resistance in sows and enhances offspring survival by modulating the gut microbiota and amino acid metabolism.

**Supplementary Information:**

The online version contains supplementary material available at 10.1186/s40104-024-01046-z.

## Background

During late gestation and lactation, sows often experience insulin resistance due to obesity or a disrupted gut microbiota [1, 2]. In an insulin-resistant state, blood glucose concentrations are elevated, inhibiting the feeding center, and delaying feeding behavior initiation [3]. This disrupts feeding continuity, leading to reduced food intake during lactation, and ultimately lower milk production in sows [4]. Studies indicate that inadequate lactation in sows during the perinatal period is a primary cause of increased piglet mortality rates [5, 6]. Piglets that consume less than 400 g of colostrum exhibit higher mortality rates within 21 d post-birth [7]. In particular for contemporary high-yield sows, low feed intake and decreased milk production are prevalent issues during lactation [8]. Therefore, alleviating insulin resistance during late gestation and lactation is crucial to enhance lactation performance in sows. Several strategies have been used to address insulin resistance in late-pregnancy and lactation phases in sows. For instance, supplementing diets with soluble fiber or resveratrol has shown promise in alleviating periparturient insulin resistance by modulating the gut microbiota [9, 10]. However, the conclusions from these studies are inconsistent. What is certain is that a modulated gut microbiota is an effective way to improve insulin resistance in sows [11].

In recent years, as probiotics have continued to gain prominence in improving the intestinal microbiota, and also the body’s immunity, anti-inflammatory, and antioxidant capacities, they have also been shown to improve reproductive performance and health status in sows [12–15]. A recent study showed that the gut microbiota had important roles regulating feed intake during lactation, while sows with high and low feed intake had a unique microbial community [11]. Emerging evidence now suggests that the gut microbiota in sows is associated with feeding efficiency [16]. Sows fed a *Bacillus subtilis* C-3102 probiotic diet (5 × 10^5^ colony forming units (CFU)/g of gestation feed and 10^6^ CFU/g of lactation feed) increased their feed intake during lactation [12]. Additionally, probiotic supplementation to sow diets affected gut microbiota composition in offspring piglets, and demonstrated potential benefits on intestinal health and growth performance [12, 17]. Previous studies also showed that *Lactobacillus reuteri* I5007 supplementation (10^9^ CFU/kg) from late gestation to lactation accelerated fecal microbiota maturation in piglets during early life and improved their growth performance [17].

*Lactobacillus rhamnosus* GG (LGG), isolated from healthy human feces, exhibits gastric acid and bile salt tolerance, and strong colonization capabilities in the gut [18, 19]. Studies in newborn and weaned piglets have shown that oral LGG benefited intestinal barrier function [20, 21]. Recently, several clinical and mouse model type 2 diabetes studies reported that LGG improved insulin resistance by modulating the gut microbiota [22–25]. However, it remains unclear whether LGG alleviates lactation performance by improving insulin resistance in sows during late pregnancy. Therefore, in this study, we hypothesized that LGG supplementation in sows improved insulin resistance by regulating the gut microbiota, thereby improving feed intake, milk yield, and piglet survival.

In this study, we (1) assessed the beneficial effects of LGG during late pregnancy and lactation on sow insulin sensitivity, lactation performance, the gut microbiota, and survival rates in offspring piglets; and (2) identified potential mechanisms whereby LGG modulated the gut microbiota and serum metabolites to alleviate insulin resistance.

## Materials and methods

### LGG preparation

LGG (ATCC 53103) was purchased from the China Center of Industrial Culture Collection and cultured in Man Rogosa Sharpe medium at 37 °C for 24 h under anaerobic conditions until the logarithmic phase, then centrifuged at 5,000 × *g* for 15 min at 4 °C, washed three times in sterile saline, and finally resuspended in saline. Our remit was to ensure that every sow in the experimental group was fed 5 × 10^10^ CFU of LGG. Sows in the control group received the same volume of saline. LGG dosages were calculated according to Wan et al. [26] and Reagan-Shaw et al. [27].

### Experimental design and feeding management

Twenty healthy third-parity sows (Landrace × Yorkshire; at d 60 of gestation) with similar genetic backgrounds were randomly assigned to 2 groups based on back-fat thickness and body weight (Table S1). Dietary treatments included a basal diet (CON; *n* = 10) and a basal diet supplemented with 5 × 10^10^ CFU/d LGG (LGG, *n* = 10). However, two sows in the CON group had miscarriages in late gestation and were ultimately not included. To maximize live probiotic intake, a pre-trial training period was initiated 2 weeks prior to official study commencement. During this period, a specialized feeding device containing a liquid solution with glucose was used to train sows to drink before their evening meal. This regimen ensured that all sows proficiently used water-sucking devices for optimal probiotic intake in 2 weeks. The sow basal diet (Table 1) was formulated to meet or exceed the nutrient requirements for both gestating and lactating animals (NRC, 2012) [28]. Animal procedures were approved by the Institutional Animal Care and Use Committee of Sichuan Agricultural University. Sows were housed in individual pregnancy crates (2.0 m × 0.60 m) from d 60 of gestation. On d 107, sows were transferred to farrowing cages (2.0 m × 2.5 m). After farrowing, newborn piglets in the same treatment group were cross-fostered within 24 h and adjusted to 10 piglets/litter. Gestational diets were provided twice daily at 08:00 h and 16:00 h. During gestation, sows were fed 2.6 kg/d on d 60 and this was stepped up to 2.75 kg/d on d 84. Sows were not fed on the day of parturition. On the second day, sows were fed 1.5 kg/d, and the amount of feeding was increased by 1.0 kg/d until they were fed ad libitum on d 4 of lactation. Before formal study commencement, all sows were fed the same basal diet. Water was freely accessible during the study.

**Table 1:** Ingredient and nutrient composition of experimental diets (as-fed basis)

Items	Gestation	Lactation
Ingredients, %		
Corn	57.79	51.17
Wheat bran	14.00	10.00
Rice bran	8.00	4.00
Soybean meal	11.00	15.45
Soybean hull	5.06	-
Soybean oil	0.55	2.53
Soybean, heat-treated	-	8.00
Fish meal (Peru)	-	2.00
Sucrose	-	2.00
Glucose	-	2.00
L-Lysine HCl (78.8%)	0.16	0.13
DL-Methionine (98%)	0.04	-
L-Threonine (98%)	0.11	0.02
L-Tryptophan (98%)	0.03	-
L-Valine (99%)	-	0.08
Dicalcium phosphate	1.22	0.89
Limestone	1.12	0.89
Sodium chloride	0.40	0.40
Premix^a^	0.52	0.44
Total	100	100
Calculated analysis		
DE, Mcal/kg	12.77	14.24
ME, Mcal/kg	11.87	13.13
Crude protein, %	14.22	18.21
Calcium, %	0.70	0.66
Phosphorus, %	0.81	0.71

^a^The premix (per kilogram of diet) provided the following: Gestation: Fe, 95 mg; Cu, 46 mg; Zn, 100 mg; Mn, 54 mg and Se, 0.17 mg. vitamin A, 6,000 IU; vitamin E, 50 IU; vitamin D_3_, 1,200 IU; vitamin K_3_, 2.4 mg; vitamin B_1_, 1 mg; vitamin B_2_, 3.6 mg; vitamin B_6_, 1.8 mg; vitamin B_12_, 0.0125 mg; biotin, 0.24 mg; folic acid, 2 mg; niacin, 25 mg; pantothenic acid, 14 mg; preservative, 500 g; antioxidant, 200 g; mycotoxin adsorbent, 1,000 g; and choline chloride (60%), 1,300 g. Lactation: Fe, 100 mg; Cu, 25 mg; Zn, 125 mg; Mn, 35 mg; I, 0.2 mg; and Se, 0.3 mg. Vitamin A, 6,000 IU; vitamin E, 50 IU; vitamin D_3_, 1,200 IU; vitamin K_3_, 2.4 mg; vitamin B_1_, 1 mg; vitamin B_2_, 3.6 mg; vitamin B_6_, 1.8 mg; vitamin B_12_, 0.0125 mg; biotin, 0.24 mg; folic acid, 2 mg; niacin, 25 mg; pantothenic acid, 14 mg; preservative, 500 g; antioxidant, 200 g; mycotoxin adsorbent, 1,000 g; and choline chloride (60%), 1,000 g

### Sample collection

Fasting blood samples were collected on d 90 and 105 of gestation, and d 10 and 21 of lactation (morning). Rectal swabs from sows were collected on d 105 of gestation and d 10 and 21 of lactation. On these latter days, litters were weighed, and piglets that were representative of the average body weight were selected from litters and blood and fresh feces were collected. Rectal swabs from piglets were collected according to Wang et al. [17]. Briefly, a sterile swab was inserted approximately 1–1.5 cm into the anal canal, moved from side to side, and left for 10–30 s to allow for microbe absorption onto the swab. Fecal samples were quickly placed in liquid nitrogen and subsequently transferred to −80 °C for storage. Colostrum was collected from sows within 3 h of parturition and used to determine milk composition. The procedure involved sterilizing the breast area with 75% alcohol, after which a representative 5 mL milk sample was collected in sterile tube and stored at –20 °C.

### Sow and piglet performance and predicted milk yields

Feed intake of sows was recorded on d 7, 14, and 21 of lactation, while piglet weight was recorded on d 7, 14, and 21 of lactation. Milk yields were predicted based on daily litter weight gain and litter size according to Hansen et al. [29].

### Serum glucose and amino acid concentration measurement

Sow fasting glucose concentrations were measured using an automatic biochemical analyzer (Hitachi 7020, Hitachi High-Tech Company, Tokyo, Japan), while serum amino acid concentrations were similarly determined using the Hitachi L-8800 model (Hitachi High-Tech).

### Enzyme-linked immunosorbent assay (ELISA)

Serum insulin, adiponectin, immunoglobulin G (IgG), immunoglobulin M (IgM), interleukin-6 (IL-6), IL-10, tumor necrosis factor-α (TNF-α), kynurenine, and indoleamine 2, 3-dioxygenase (IDO) concentrations were determined using respective ELISA kits (Meimian Biotechnology, Jiangsu, China) according to manufacturer’s protocols.

### DNA extraction and 16S rRNA gene sequencing

Microbial genomic DNA from fecal samples was extracted using QIAamp DNA Stool Mini kits (Qiagen Inc., Hilden, Germany) according to the manufacturer’s instructions. Extracted DNAs were checked on 1% agarose gels, and concentrations and purity were determined using a NanoDrop 2000 spectrophotometer (Thermo Scientific, USA). Based on concentrations, DNAs were diluted to 1 ng/µL in sterile water and used as templates for PCR. The V3–V4 hypervariable region of 16S rRNA was amplified using 341F (5´-CCTAYGGGRBGCASCAG-3´) and 806R (5´-GGACTACHNGGGTATCTAAT-3´) primers. Briefly, PCR reactions were carried out in 30 µL reactions, and contained 15 µL of Phusion^®^ High-Fidelity PCR Master Mix (New England Biolabs), 0.2 µmol/L each of forward and reverse primers, and approximately 10 ng of template DNA. Thermal cycling conditions consisted of initial denaturation at 98 °C for 1 min, followed by 30 denaturation cycles at 98 °C for 10 s, annealing at 50 °C for 30 s, and elongation at 72 °C for 30 s. Finally, the program was maintained at 72 °C for 5 min. PCR products were purified using Qiagen Gel Extraction kits (Qiagen Inc., Hilden, Germany) after electrophoresis on 2% gels containing SYBR green. Amplicons were quantified, pooled, and sequenced using the Illumina MiSeq system (Illumina Inc., San Diego, CA, USA) for paired-end reads. For bioinformatics analysis, raw paired-end reads were merged using FLASH (v. 1.2.7), and then high-quality clean tags were obtained by quality filtering raw tags, following QIIME (v. 1.9.1) quality-control processes. Sequence analysis was conducted using Uparse software (v. 7.0.1001), grouping sequences with ≥ 97% similarity into the same operational taxonomic units (OTUs) and selecting representative sequences for further annotation. OTU abundance information was normalized to the sequence number of the sample with the fewest sequences, and subsequent α and β diversity analyses were conducted using this normalized data. Alpha (α) diversity was used to analyze species diversity in samples using three indices: Observed-species, Chao1, and Ace. All samples indices were calculated using QIIME (v. 1.7.0) and displayed using R software (v. 2.15.3). Beta (β) diversity analyses were used to assess differences in sample species complexity, with both weighted and unweighted UniFrac distances calculated using QIIME software (v. 1.9.1). Principal coordinate analysis (PCoA) based on Bray–Curtis distances was used to visualize sample differentiation or similarity and groups were deemed significantly distinct using ANOSIM analysis in R software (v. 2.15.3). Linear discriminant analysis effect size (LEfSe) was used to detect differentially abundant features across groups in R software (v. 1.0). PICRUSt analysis was used to predict microbial community functions based on 16S rRNA gene sequencing in R software (v. 4.0.3).

### Short chain fatty acid (SCFA) determination

SCFAs were determined according to Li et al. [30]. Briefly, fecal samples were thawed at 4 °C and approximately 50 mg of a uniform, representative sample was diluted in 1 mL of ultrapure water and centrifuged at 3,500 × *g* for 15 min. After, the supernatant was collected and mixed with 0.2 mL of metaphosphoric acid (25%) and 23.3 µL of crotonic acid (210 mmol/L). The mixture was placed at 4 °C for 30 min before centrifugation at 4,000 × *g* for 10 min. Finally, 0.9 mL of methanol was mixed with 0.3 mL of the supernatant and filtered through a 0.22-µm filter (Millipore Co., Bedford, MA, USA) after centrifugation at 3,500 × *g* for 5 min. Gas chromatography (Varian CP-3800, manual injection, flame ionization detector, FID, 10 µL micro-injector) was used to determine SCFA concentrations.

### Untargeted serum metabolomics

Frozen serum samples were thawed at 4 °C, after which 100 µL was mixed with 400 µL of cold methanol/acetonitrile (1:1, v/v) to remove protein. The mixture was centrifuged for 20 min at 4 °C and then dried in a vacuum centrifuge. Samples were then dissolved in 100 µL of acetonitrile/water (1:1 volume ratio) and centrifuged at 14,000 × *g* for 15 min at 4 °C. Supernatants were then analyzed using Ultra-High Performance Liquid Chromatography (UHPLC, 290 Infinity LC, Agilent Technologies) coupled to a quadrupole time-of-flight (AB Sciex TripleTOF 6600) at Shanghai Applied Protein Technology Co., Ltd.

Raw data were converted to mzXML files in ProteoWizard. Peak picking was performed using the following parameters: centwave *m/z* = 10 ppm, peak-width = c (10, 60), and prefilter = c (10, 100). Peak grouping was performed using bw = 5, mzwid = 0.025, and minfrac = 0.5. The collection of metabolite profile annotation algorithms facilitated isotope/adduct annotation. Only variables with > 50% non-zero values in at least one group were retained in extracted ion features. Metabolite identification relied on accurate *m/z* values (< 10 ppm) and comparisons with Tandem Mass Spectrometry (MS/MS) spectra in an in-house database of authenticated standards. Metabolite structures were confirmed by matching accurate mass numbers (within < 25 ppm) and using a secondary spectrogram matching method and the laboratory database at Shanghai Applied Protein Technology Co. Ltd. Processed data were analyzed using the R package, where they underwent multivariate data analysis, including Pareto-scaled principal component analysis and orthogonal partial least-squares discriminant analysis (OPLS-DA). Variable importance in projection (VIP) values of each variable in the OPLS-DA model were calculated to indicate their contribution to the classification. Student’s *t*-tests were used to determine significant differences between two groups of independent samples. VIP > 1 and *P-*values < 0.05 were used to screen for significant metabolite changes. For differential metabolite analysis, we used Human Metabolome Database (HMDB) and Kyoto Encyclopedia of Genes and Genomes (KEGG) databases. Furthermore, all differentially abundant metabolites were cross-referenced to KEGG (http://www.kegg.jp/) and mapped to KEGG pathways.

### Statistical analysis

Data were analyzed using SAS 9.4 (SAS Inst. Inc., Cary, NC, USA) with individual sows or piglets as experimental units. Serum indicators including glucose, insulin, Homeostatic Model Assessment for Insulin Resistance/Insulin Sensitivity (HOMA-IR/IS), and adiponectin (ADP) were analyzed using the PROC MIXED procedure in SAS for repeated measurements in a completely randomized design using the following models:

$$Y_{ij}= \mu +\alpha_{i}+e_{ij}$$

$$Y_{ijk} = \mu + \alpha_{i} + \beta_{j} + \alpha_{i} \times \beta_{j} + e_{ijk}$$

where *Y* (*Y*_*ij*_, *Y*_*ijk*_) is an observed trait, *µ* is the population mean, *α*_*i*_ is the fixed effect of the treatment (*i* = CON or LGG), *β*_*j*_ is the fixed effect of gestation stage (*j* = G90, G105, L10, or L21), *α*_*i*_ × *β*_*j*_ is the interaction between diet and gestation stage, and *e* (*e*_*ij*_, *e*_*ijk*_) is the residual, which was assumed to be normally distributed with variance homogeneity. Piglet survival rates were analyzed using Chi-square tests. Gut microbiota composition was analyzed using Wilcoxon tests, with *P*-values corrected using the Benjamini–Hochberg method. Other data were subjected to Student’s *t*-tests in a completely randomized design using SAS and were presented as the mean ± standard error of the mean (SEM). Significant differences were accepted at *P* < 0.05 and tendencies at 0.05 ≤ *P* < 0.10.

## Results

### LGG supplementation enhances lactation performance and alleviate sow insulin resistance

**Fig. 1:**
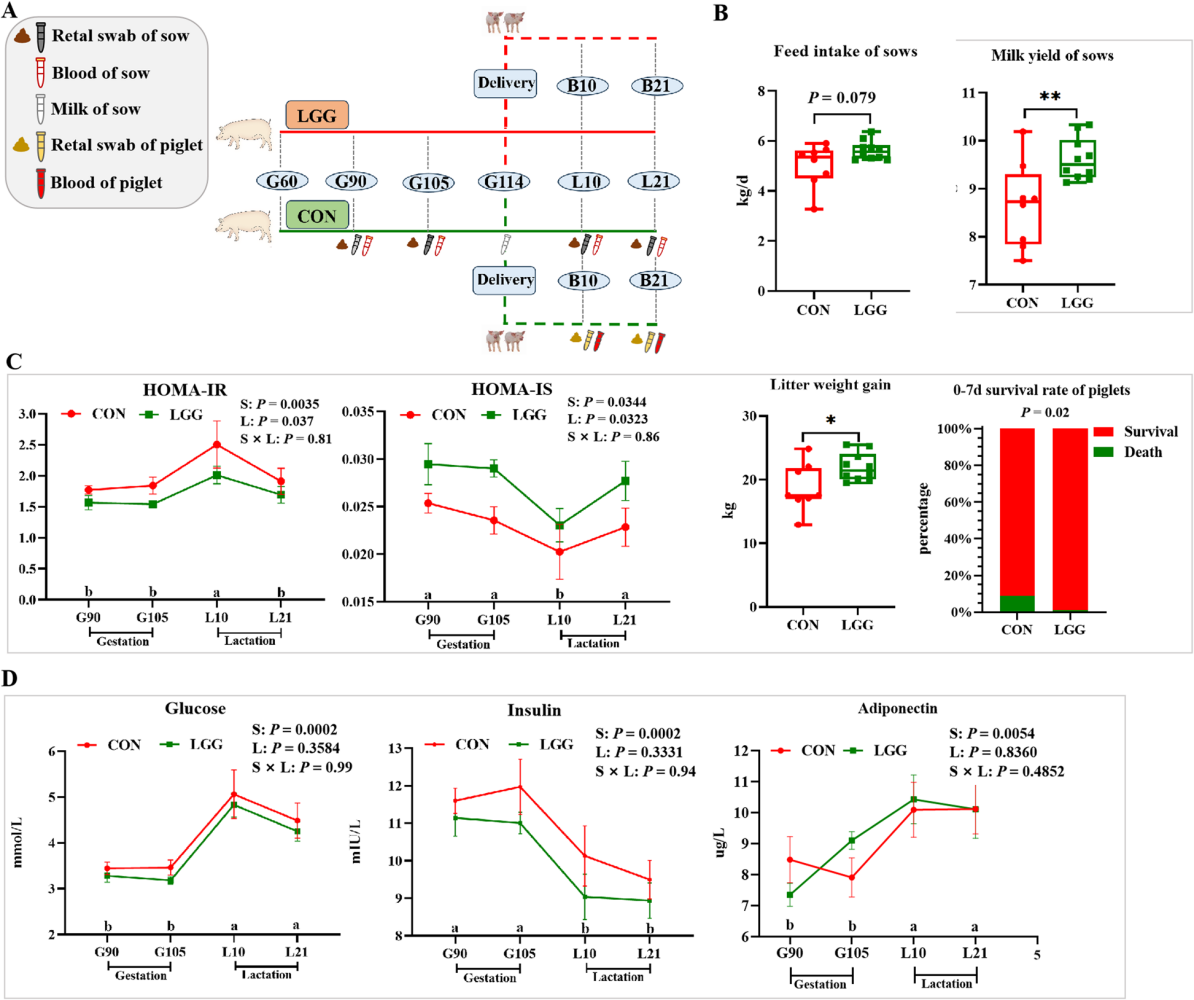
The overall trial design and LGG supplementation effects on insulin resistance and performance in sows. LGG was supplemented from d 60 of gestation until the end of lactation. Serum and fecal samples were collected on d 90 (G90), 105 (G105) of gestation, and days 10 (L10) and 21 (L21) of lactation. Milk was collected on the day of parturition (G114), and piglet samples were collected on d 10 and 21 of lactation (**A**). Changes in sow feed intake, lactation yield, litter weight gain, and piglet survival after LGG supplementation (**B**). Comparison of the insulin sensitivity index between sow groups during late gestation and lactation (**C**). Changes in serum glucose, insulin, and adiponectin concentrations in sows (**D**). S: stage, L: LGG, S × L: interaction between reproductive stage and LGG treatment. Serum biochemical indicators, including glucose, insulin, HOMA-IR, HOMA-IS, and adiponectin were analyzed using the PROC MIXED procedure (SAS) for repeated measurements in a completely randomized design. Piglet survival rates were analyzed using Chi-square tests. Other data were analyzed using Student’s *t*-tests. HOMA: homeostasis model assessment. IR: insulin resistance. IS: insulin sensitivity. Data were presented as the mean ± SEM. ^*^P < 0.05 and ^**^P < 0.01. Tendencies were accepted at 0.05 ≤ *P* < 0.10

**Fig. 2:**
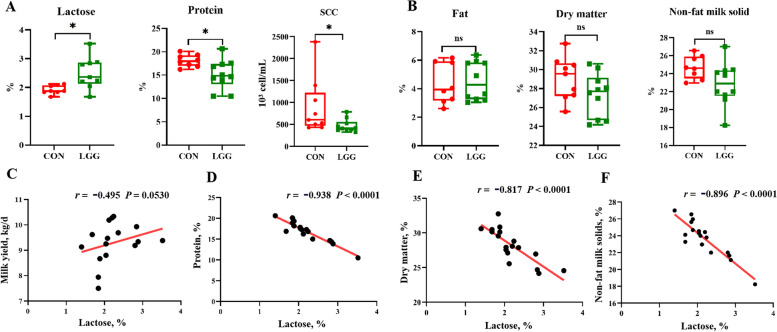
LGG supplementation to sows improves milk yield and is associated with increased lactose levels. Comparison of lactose, protein, somatic cell counts (SCC), fat, dry matter, and non-solid milk (**A** and **B**). Linear regression analysis of lactose content in colostrum (*X*) and milk yield (*Y*), non-solid milk (*Y*), protein (*Y*) and dry matter (*Y*) (**C**–**F**). The student’s *t*-test was used to compare differences between sow groups. Data were presented as the mean ± SEM. Correlations between lactose and milk yield, non-solid milk, protein, and dry matter were analyzed using Pearson’s correlation analysis

To explore the effects of LGG on lactation performance in sows, blood and fecal samples were collected on gestational d 90 and 115 and lactation d 10 and 21. Milk samples were collected on the day of parturition. Blood and fecal samples from offspring piglets were collected at the same time points as the sows (Fig. 1A). Sow lactation performance is shown (Fig. 1B). The average daily feed intake in LGG-supplemented sows showed a marginal increase of approximately 500 g/d when compared to the CON group (*P* = 0.079). Notably, during the first lactation week, milk yields in the LGG group significantly surpassed CON levels (*P* < 0.01; Fig. 1B). Concurrently, heightened survival rates and increased litter weights of newborn piglets were observed in this time frame. Across the entire lactation period, feed intake, milk yields, and litter weight showed no statistically significant differences (*P* > 0.05; Fig. S1A). However, piglet survival rates in the LGG group remained significantly improved (*P* < 0.01; Fig. S1B). Furthermore, no significant differences in body weight or back-fat thickness were observed between groups (*P* > 0.05; Fig. S1C). LGG sows showed higher (*P* < 0.05; Fig. 1C) insulin sensitivity than CON sows during late gestation and lactation periods. Sows showed higher insulin sensitivity at lactation on d 10 (L10) than at gestation day 90 (G90), G105, and L21 (*P* < 0.05; Fig. 1C). However, no significant interaction effects were noted between time and dietary treatments (*P* > 0.05). Additionally, LGG increased lactose and decreased milk protein levels, and also reduced somatic cell counts (SCC) in colostrum when compared to CON sows (*P* < 0.05; Fig. 2A). However, no significant differences were observed in milk fat, dry matter, or non-fat milk solids between groups (*P* > 0.05; Fig. 2B). Pearson’s correlation analysis revealed a positive correlation between lactose and milk yields (*r* = 0.495, *P* = 0.053; Fig. 2C), and strong negative correlations between lactose and milk proteins (*r* = −0.983, *P* < 0.0001; Fig. 2D), dry matter (*r* = −0.817, *P* < 0.0001; Fig. 2E), and non-solid milk content (*r* = −0.896, *P* < 0.0001; Fig. 2F).

### LGG supplementation reshapes fecal microbiota composition in sows during late gestation and lactation periods

**Fig. 3:**
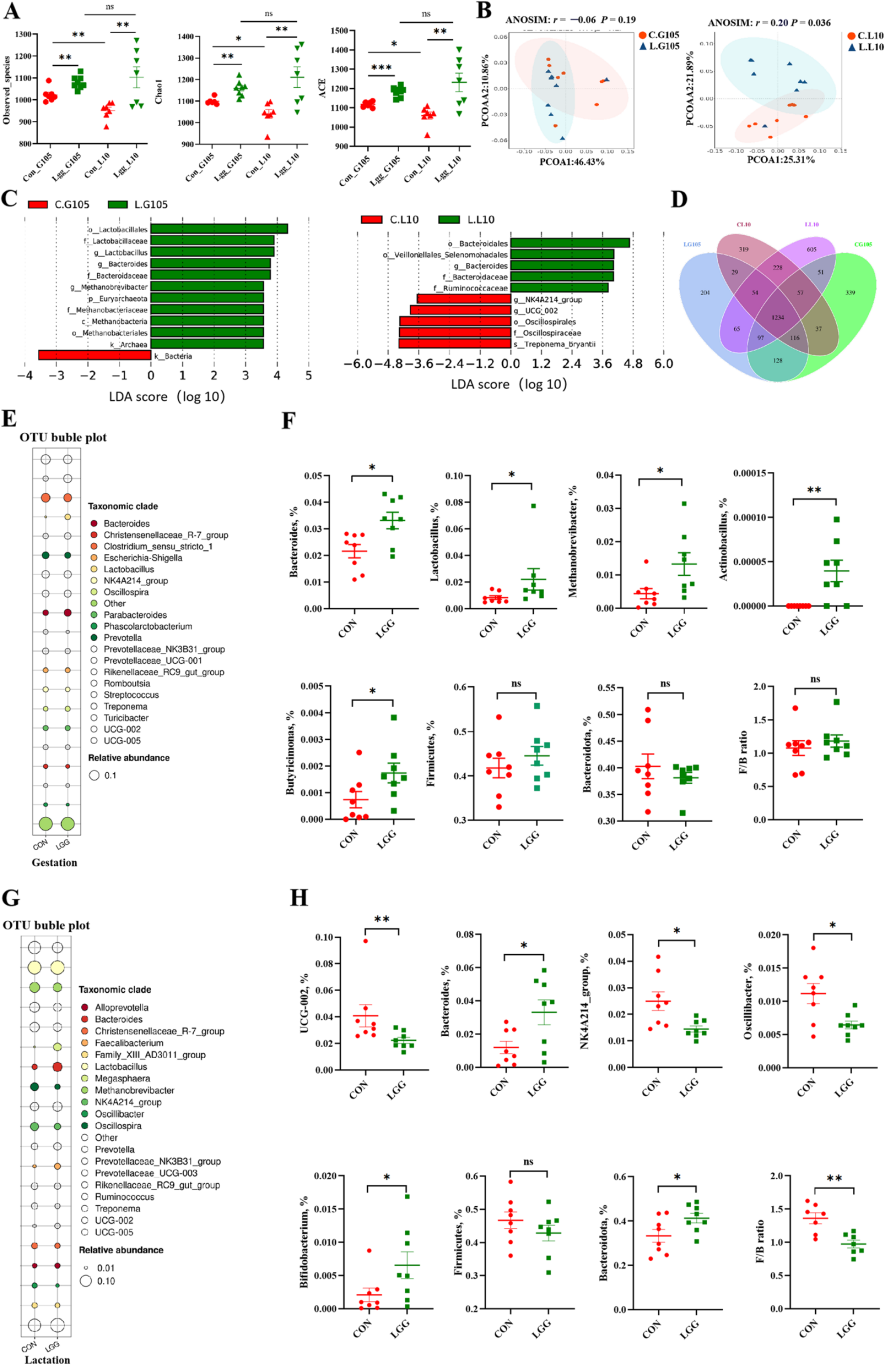
LGG supplementation reshapes the gut microbiota in sows during late gestation and lactation. Gut microbiota α diversity, including observed species, ACE, and Chao 1 indices (**A**). Principal coordinates analysis (PCoA) score plots (**B**). LefSe bar (**C**) and Venn diagram (**D**) showing sows on d 105 of gestation (G105) and d 10 of lactation (L10). OTU bubble maps were drawn for the top 20 bacteria ranked by relative abundance at G105 and L10 (**E** and **G**). The relative abundance of the significantly different top 5 genera at G105 and L10 (**F** and **H**). Data were presented as the mean ± SEM. PCoA ordination plots show fecal bacterial communities in CON and LGG groups based on Bray–Curtis distances. Groups were deemed significantly distinct using ANOSIM analysis (*P* < 0.05). Wilcoxon tests were used to evaluate statistical differences. ^*^P < 0.05, ^**^P < 0.01 and ns means not significant (*P* > 0.05)

As shown in Fig. 3A, the microbiota α diversity index in feces from LGG-supplemented sows in gestation and lactation periods was significantly higher (*P* < 0.05) when compared to CON sows. At the same time, a significant reduction (*P* < 0.05; Fig. 3A) in α diversity in transition from post-pregnancy to lactation in CON sows was observed; however, this phenomenon was not observed in LGG sows. PCoA using Bray–Curtis dissimilarity analyses distinctly separated LGG from CON sows during both gestation and lactation stages (*P* < 0.05; Fig. 3B). In addition, LEfSe analysis, which used to identify biomarkers across groups, revealed significant increases (*P* < 0.05; Fig. 3C) in genera abundance (*Lactobacillus, Bacteroides*, and *Methanobrevibacter*) due to LGG supplementation during late gestation. Furthermore, during mid-lactation, a notable increase in *Bacteroides* abundance was attributed to LGG (*P* < 0.05; Fig. 3C). To further investigate differential flora levels between groups, we generated an OTU bubble plot which clearly showed the top 20 genera and their relative abundance across groups. Venn diagram data showed that LGG and CON groups shared 128 OTUs in late gestation and 228 OTUs in lactation periods (Fig. 3D). *Bacteroides* were predominant in both late gestation (Fig. 3E) and lactation periods (Fig. 3G). Wilcoxon rank sum tests were then used to explore differential microorganisms between groups; the top 5 significantly different bacteria are shown (Fig. 3). Relative *Bacteroides*, *Lactobacillus*, *Methanobrevibacter*, *Actinobacillus*, and *Butyricimonas* abundance at the genus level was higher (*P* < 0.05; Fig. 3F) in LGG sows when compared with CON sows at G105. However, no significant differences (*P* > 0.05; Fig. 3F) in relative Firmicutes and Bacteroidota abundance, and their ratios (F/B) were observed. At L10, the top 5 dominant genera were *Ucg-002, Bacteroides, NK214_group, Oscillibacter*, and *Bifidobacterium* (*P* < 0.05; Fig. 3H). Additionally, increased *Bacteroidetes* abundance and decreased F/B (*P* < 0.05; Fig. 3H) were observed following LGG supplementation.

**Fig. 4:**
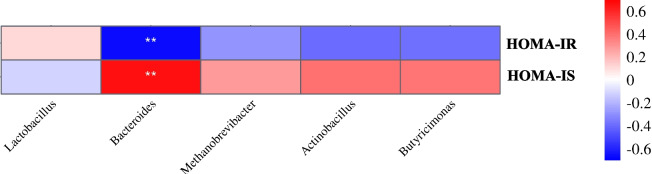
Serum insulin resistance is associated with *Bacteroides* abundance in late gestating sows. Spearman’s correlation analysis showed that the serum insulin resistance index at d 105 was strongly associated with intestinal *Bacteroides* abundance. HOMA: Homeostasis Model Assessment. IR: insulin resistance. IS: insulin sensitivity. Red squares indicate a positive correlation and blue squares indicate a negative correlation. ^*^P < 0.05 and ^**^P < 0.01

To explore whether insulin resistance was associated with gut microbiota changes, we performed Spearman’s correlation analyses between the insulin resistance index and differential bacteria (top 5 genera). Correlation analyses showed that insulin resistance at G105 was significantly associated with *Bacteroides* abundance in feces (*r* = −0.66, *P* < 0.05; Fig. 4). We also examined SCFAs, including acetic acid, propionic acid, butyric acid, isobutyric acid, valeric acid, and isovaleric acid levels, in sow feces at G105. However, no significant differences were observed between groups (*P* > 0.05; Table S2). Thus, LGG supplementation during late gestation significantly reshaped gut microbiota composition in sows and was associated with insulin resistance.

### LGG supplementation alters serum metabolites at G105

**Fig. 5:**
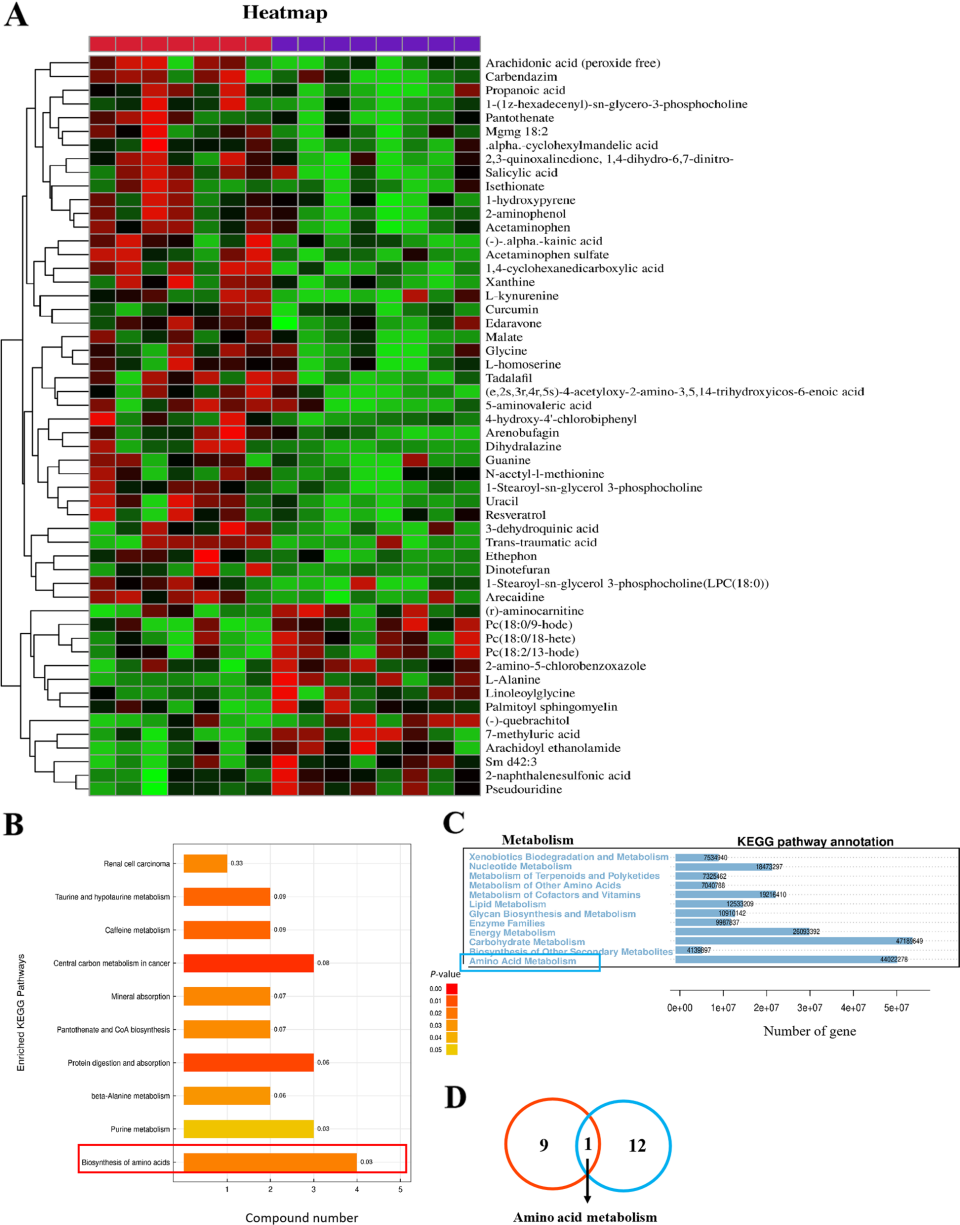
LGG supplementation alters serum metabolites in late gestating sows. Cluster plots show 49 significantly different serum metabolites between LGG and CON groups (**A**). Serum metabolites in KEGG pathway analysis (**B**) and microbial 16S rRNA function predictions (**C**) show that amino acid supplementation of LGG significantly changed amino acid metabolism pathway. Amino acid metabolism was a shared KEGG pathway among the 2 omics (**D**)

**Fig. 6:**
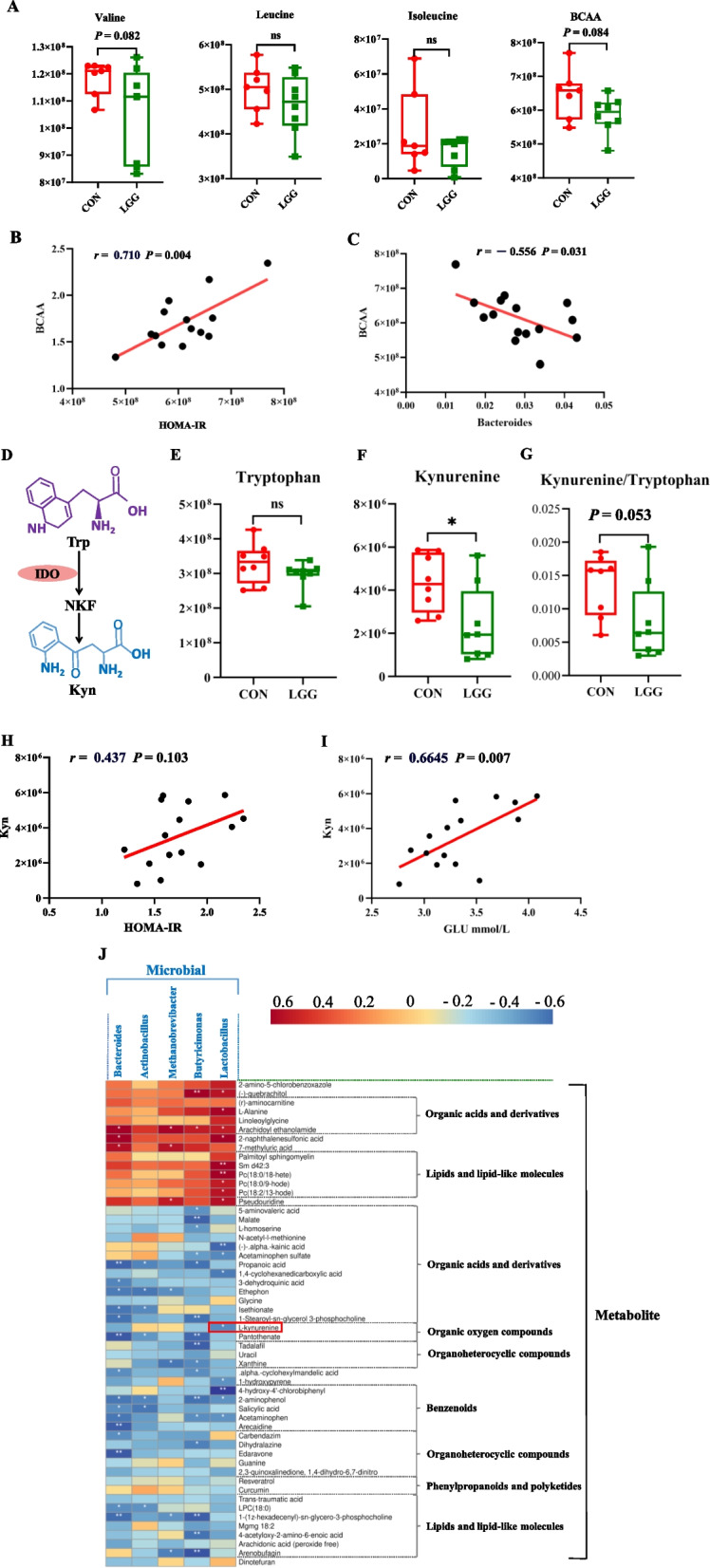
LGG improves insulin resistance in late gestating sows by regulating the gut microbiota, branched-chain amino acid (BCAA), and kynurenine levels. BCAA levels in serum metabolites (**A**). Pearson’s correlation analysis of BCAAs with the insulin resistance index HOMA-IR (**B**) and Bacteroides (**C**). The kynurenine production pathway (**D**), tryptophan (**E**), kynurenine (**F**) levels in the metabolome and their ratios (**G**). Correlation analysis showing serum kynurenine content with HOMA-IR (**H**) and glucose (**I**). Spearman’s correlation analysis showed a correlation between differential microbial features and serum metabolites in sows at 105 days of gestation (**J**). BCAA: branched chain amino acids. Trp: tryptophan. Kyn: kynurenine. IDO: indoleamine 2,3-dioxygenase. GLU: glucose. HOMA: homeostasis model assessment. IR: insulin resistance. ^*^P < 0.05, ^**^P < 0.01 and ns refers to not significant (*P* > 0.05)

Given that LGG supplementation significantly altered the gut microbiota, we further investigated serum metabolite changes in sows. UHPLC was used to detect serum metabolites at G105. In total, 1,249 metabolites (717 and 532 under positive and negative ion modes, respectively) were detected across groups. A hierarchical clustering heatmap of significant differential metabolites showed differences in metabolite expression patterns in different samples (*P* < 0.05; Fig. 5A). A volcano plot showed 231 (130 and 101 up- and down-regulated peaks, respectively) and 279 (155 and 124 up- and down-regulated peaks, respectively) peak features in positive and negative ion modes, respectively (Fig. S2A). An OPLS-DA score plot also showed different serum metabolic profiles in CON and LGG groups (Fig. S2B). Using VIP > 1 and *P* < 0.05 parameters, 54 significant metabolite changes were identified between groups (26 and 28 under positive and negative ion modes, respectively), of which, 14 metabolites were enriched in LGG sows, and the remaining were enriched in CON sows. KEGG pathway analyses showed that 10 metabolic pathways were altered, and four metabolites were significantly enriched in the amino acid biosynthesis pathway (*P* < 0.05; Fig. 5B). Then, PICRUSt analysis was used to predict microbial community functions based on 16S rRNA sequencing (Fig. 5C), including metabolic pathway and KEGG enrichment analyses. We observed that amino acid metabolism was a significantly enriched metabolic pathway shared by the serum metabolome and fecal microbial function predictions (Fig. 5D). Further data analyses showed that serum branched-chain amino acids (BCAA) including valine, leucine, and isoleucine tended (*P* = 0.0844; Fig. 6A) to be decreased following LGG supplementation. Correlation analysis also revealed a significant positive correlation between BCAA and insulin resistance (*r* = 0.710, *P* < 0.001; Fig. 6B). Conversely, a significant negative correlation was observed between BCAA and *Bacteriodes* abundance (*r* = −0.556, *P* < 0.05; Fig. 6C). Tryptophan is metabolized by IDO to produce kynurenine (Fig. 6D). In our study, there was no significant difference in tryptophan levels between the two sow groups (*P* > 0.05; Fig. 6E). However, serum metabolome data analyses revealed a significant reduction in serum kynurenine content following LGG supplementation (*P* < 0.05; Fig. 6F). The kynurenine to tryptophan ratio, which is used to measure IDO content, showed a decreasing tendency (*P* = 0.053; Fig. 6G) following LGG supplementation. Serum kynurenine did not show a significant positive correlation with insulin resistance (*r* = 0.437, *P* = 0.103; Fig. 6H). However, a significant positive correlation was observed between serum kynurenine and serum glucose (*r* = 0.6645, *P* < 0.01; Fig. 6I). Interestingly, heatmap analysis revealed a clear correlation between these significantly up-regulated microorganisms and metabolites (*P* < 0.05; Fig. 6J). Specifically, with the exception of *Actinobacillus*, the remaining up-regulated bacteria all showed correlations with metabolites, with the strongest correlation identified for *Lactobacillus* toward organic acids and derivatives and lipids and lipid-like molecules. Importantly, *Lactobacillus* was significantly negatively correlated with serum kynurenine (*P* < 0.05; Fig. 6J).

### LGG supplementation reduces serum BCAA and kynurenine

**Fig. 7:**
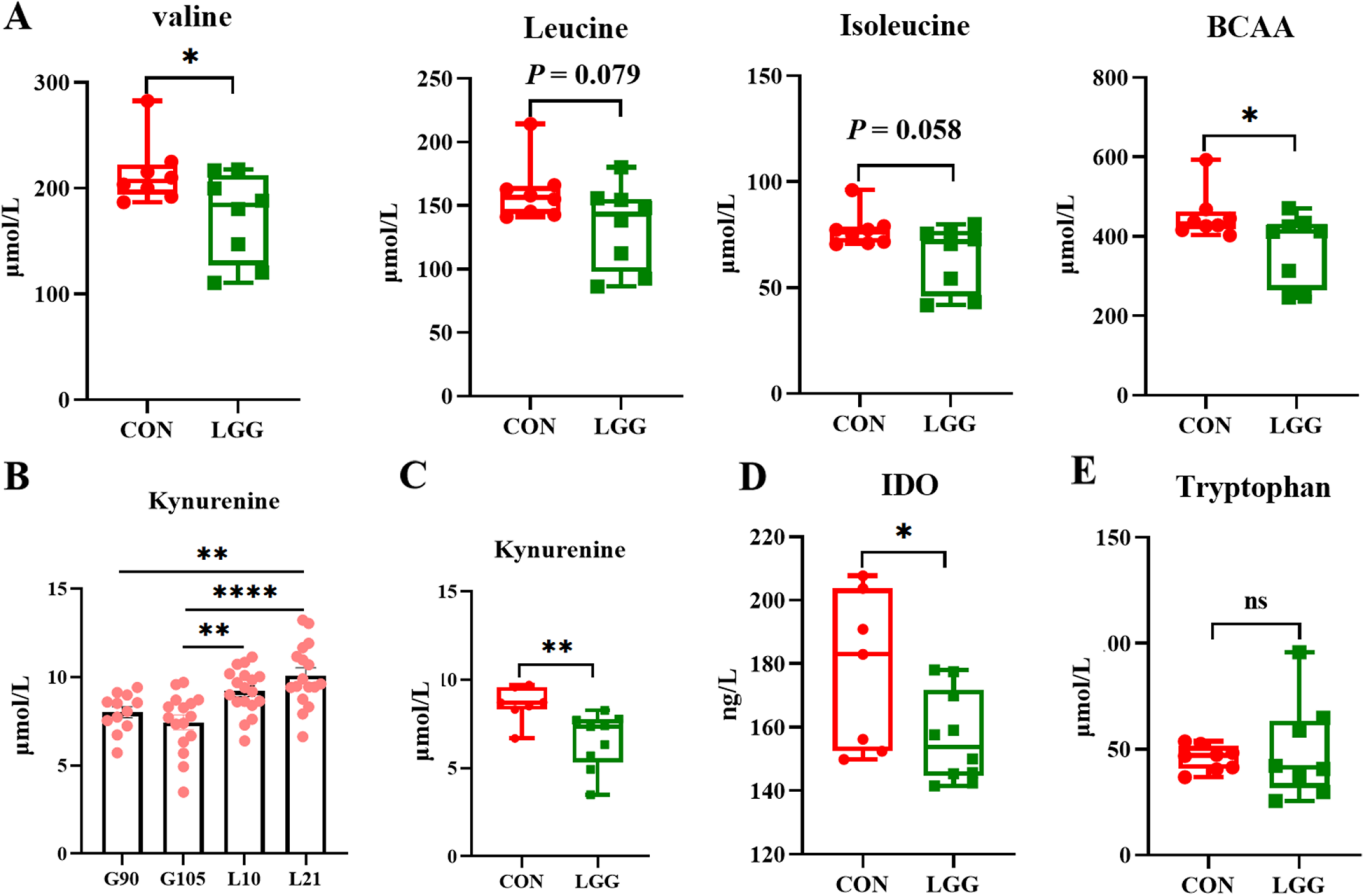
Quantitative analysis of serum branched-chain amino acid and kynurenine levels in late gestating sows. Branched-chain amino acids (valine, leucine, and isoleucine) in sow serum on d 105 of gestation (**A**). Serum kynurenine levels on d 90 and 105 of gestation and d 10 and 21 of lactation (**B**). Serum kynurenine (**C**), tryptophan (**D**), and IDO (**E**) levels on d 105 of gestation. BCAA: branched chain amino acids. IDO: indoleamine 2,3-dioxygenase. Data were analyzed using Student’s *t*-tests or one-way analysis of variance, and were presented as the mean ± SEM. ^*^P < 0.05, ^**^P < 0.01 and ns indicates not significant (*P* > 0.05)

To confirm BCAA and kynurenine reductions in serum, we quantified amino acids in serum from G105 sows. As expected, LGG supplementation significantly reduced serum BCAA levels (*P* < 0.05; Fig. 7A). Serum kynurenine levels increased with gestation progression (*P* < 0.01; Fig. 7B), whereas it was significantly decreased in LGG sows at G105 (*P* < 0.05; Fig. 7C). Serum IDO levels at G105 were also significantly decreased (*P* < 0.01; Fig. 7D) following LGG supplementation. However, LGG supplementation had no significant effects on serum tryptophan levels (*P* > 0.05; Fig. 7E) at G105, or on serum kynurenine and IDO levels (*P* > 0.05) (Fig. S3) at G90, L10, and L21.

### Maternal LGG supplementation modifies the gut microbiota in offspring piglets

**Fig. 8:**
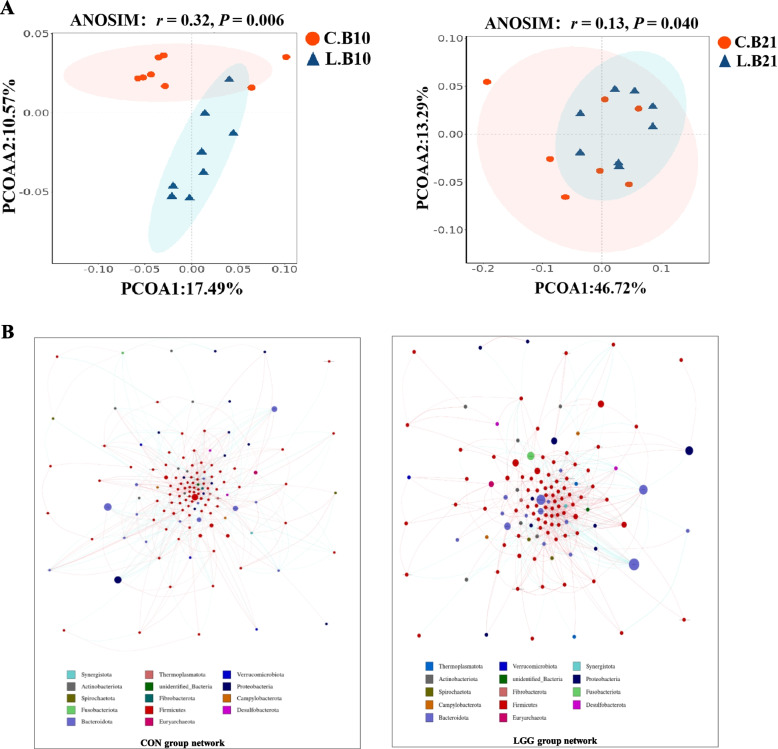
LGG supplementation in sows influences gut microbial communities in offspring piglets. Principal coordinates analysis (PCoA) score plots (**A**), microbial interaction network diagram (**B**). PCoA ordination plots show fecal bacterial communities in CON and LGG groups based on Bray–Curtis distances. Groups were deemed significantly distinct using ANOSIM analysis (*P* < 0.05)

To investigate the potential impact of maternal LGG consumption on the gut microbiota in piglet offspring, fecal samples collected at L10 and L21 underwent 16S rRNA sequencing. We observed that fecal microbiota α diversity was significantly increased (*P* < 0.05; Fig. S4A) as piglets matured, but with no significant differences (*P* > 0.05, Fig. S4A) between groups. However, PCoA revealed a more pronounced impact of maternal LGG supplementation on piglet microbial communities at L10 (*P* < 0.05; Fig. 8A). Gut microbial interaction network analyses showed that the gut microbiota at L10 had more complex structures in the LGG group (Fig. 8B), characterized by higher density, greater depth, and more nodes, when compared with CON piglets (Table S3). We further analyzed genus differences in piglet groups, and found that LGG supplementation increased the relative abundance of the beneficial bacteria *NK4A214_group*, while it decreased the relative abundance of harmful bacteria such as *Streptococcus* and *Klebsiella* (*P* < 0.05; Fig. S4B). Thus, maternal LGG supplementation conferred greater benefits toward the piglet microbiome during early lactation.

### Maternal LGG supplementation modulates immunoglobulin and inflammatory cytokines levels in sow milk and piglet serum

**Fig. 9:**
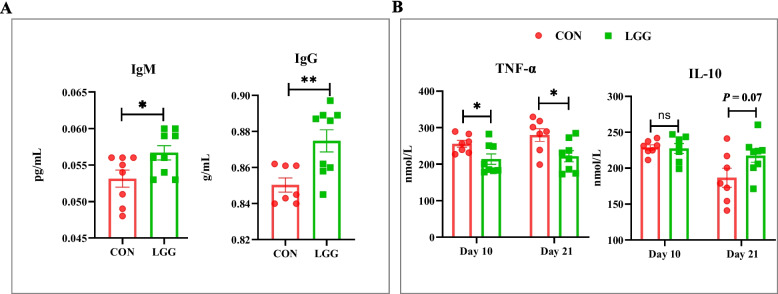
Maternal LGG supplementation improves cord blood immunoglobulins and affects piglet inflammation. Immunoglobulin M and G levels in umbilical cord blood (**A**), IL-10 and TNF-α levels in piglet serum on d 10 and 21 of lactation (**B**). Data were analyzed using Student’s *t*-tests and presented as the mean ± SEM. ^*^P < 0.05 and ^**^P < 0.01. Tendencies were declared at 0.05 ≤ *P* < 0.10. ns indicates not significant (*P* > 0.05)

To assess the impact of maternal LGG supplementation on immunoglobulins and inflammatory cytokines in sow milk and piglet serum, we analyzed several parameters (Fig. 9). LGG supplementation exerted no effects (*P* > 0.05; Fig. S5) on immunoglobulin levels in milk, whereas it generated higher (*P* < 0.05; Fig. 9A) IgG and IgM levels in umbilical cord serum. For inflammatory cytokine analysis in piglet precaval serum, TNF-α levels at L10 and L21 were lower (*P* < 0.05; Fig. 9B) in LGG piglets when compared with CON animals, whereas IL-10 levels at L21 tended (*P* = 0.07; Fig. 9B) to be higher in LGG piglets when compared with CON animals.

## Discussion

In sows, a high feed intake and high body reserve mobilization are considered key prerequisites for higher milk production [4]. However, when production cannot meet the nutritional needs of piglets, sows over-mobilize their own body reserves, resulting in reduced reproductive lives [31]. In our study, sows fed diets supplemented with LGG had higher milk production levels during the first lactation week, while body weight, body lipid, and protein loss between groups did not differ. In contrast, when compared with CON sows, LGG supplementation improved feed intake during the first lactation week. These observations suggested that LGG supplementation increased milk production by increasing food intake rather than sows mobilizing their own body reserves. Insulin resistance is one characteristic of perinatal metabolic syndrome in gestating and lactating sows [1], where excessive insulin resistance reduces feed intake during lactation [2]. We showed that insulin sensitivity was increased in sows fed LGG diets when compared with CON sows, consistent with a study showing that oral *Lactobacillus rhamnosus *LS-8 administration generated lower insulin resistance index values in high-fat diet mice [32]. Therefore, high insulin sensitivity may contribute to high food intake in LGG-fed sows during lactation. Several previous studies indicated that LGG increased insulin sensitivity by increasing adiponectin levels in mice with type 2 diabetes or insulin resistance [33–35]. However, in our study, no differences in serum adiponectin concentrations were recorded between groups, possibly owing to species-specific differences.

**Fig. 10:**
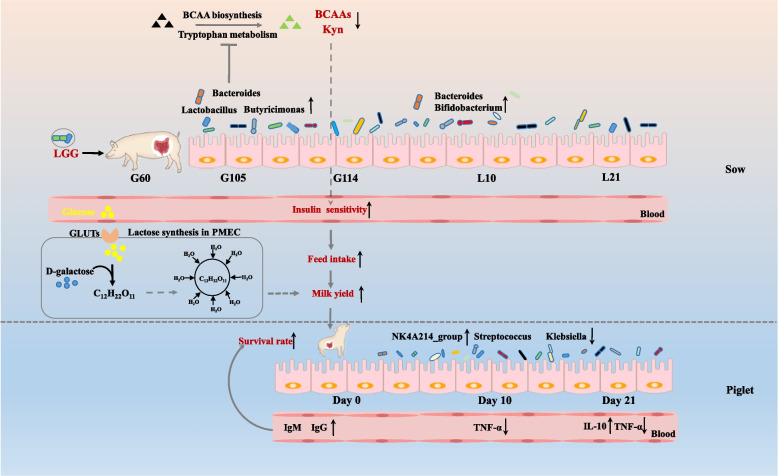
Mechanism showing LGG supplementation effects on sow performance and piglet health. In sows, LGG supplementation reshaped the gut microbiota and affected branched-chain amino acid and kynurenine synthesis in late gestation and lactation periods, and improved insulin sensitivity, feed intake, and lactation yields. This ultimately improves piglet survival in early lactation. Additionally, the beneficial effects of LGG supplementation include increased immunoglobulin levels in cord blood and improved inflammatory responses in piglets. PMEC: pig mammary epithelial cells

Another important milk yield factor is lactose [36, 37], which is not only an important nutrient, but also directly affects milk yields by regulating osmotic pressure in mammary epithelial cells [36]. In our study, increased lactose levels were observed when sows were fed an LGG-supplemented diet; a positive correlation was observed between lactose and milk yields. As expected, lactose was strongly negatively correlated with other solid components in milk, consistent with previous studies showing that the content of other solids decreased as milk yields increased [37, 38]. Circulating glucose is the only substrate source of lactose synthesis in sow mammary glands [39]. Glucose uptake is dependent on glucose transporters, with insulin-dependent glucose transporters 8 identified in mammary glands in recent years [40]. Therefore, we speculated that higher insulin sensitivity in LGG-fed sows promoted glucose uptake capacity in mammary glands, thereby synthesizing more lactose (Fig. 10). Milk is the only energy source for newborn piglets. Therefore, increased milk yields and lactose levels in LGG sows contribute to greater offspring survival rates and growth performance.

Current evidence now shows that gut microbiota dysbiosis is one cause of insulin resistance in sows [1]. As our data indicated, gut microbiota α-diversity was higher in LGG sows when compared to CON sows, consistent with research showing that LGG enhanced α-diversity in an insulin resistance mouse model [41]. Previous reports also showed that individuals with insulin resistance had lower *Lactobacillus* abundance [42, 43], which could be increased by LGG [44]. Consistent with these observations, we identified higher *Lactobacillus* abundance in LGG sows. Additionally, in patients with insulin resistance, the gut microbiota was characterized by lower *Bacteroidetes* abundance and F/B ratios [45]. In our study, lactating sows fed an LGG diet increased their *Bacteroidetes* abundance levels and F/B ratios, consistent with previous findings in mice [24]. Previous studies also showed that LGG supplementation improved *Bacteroides* abundance [23]. Interestingly, in our study, *Bacteroides* were predominant in both late gestation and lactation periods. Spearman’s correlation analyses showed a significant negative correlation between *Bacteroides* abundance and insulin resistance at late gestation. Therefore, LGG consumption appeared to improve insulin sensitivity and lactation performance in sows by modulating the gut microbiota [46].

Serum metabolites are important markers that reflect metabolic status and bodily health [47, 48]. Recent studies confirmed that a dysregulated gut microbiota influenced host amino acid metabolism and contributed to insulin resistance [49–51]. In particular, high serum BCAA levels were shown to promote insulin resistance development, correlating with reduced *Bacteroides* abundance [52, 53]. Interestingly, we demonstrated that LGG supplementation reduced serum BCAA levels in sows. Further analysis revealed a positive correlation between BCAA and insulin resistance, while a negative correlation was observed between BCAA and *Bacteroides* abundance. Thus, ameliorated insulin resistance in LGG-fed sows was potentially linked to altered BCAA levels and *Bacteroides* abundance.

Kynurenine is the main tryptophan metabolite [54]. A recent study reported a crucial role for the gut microbiota in tryptophan metabolism [55]. Notably, elevated kynurenine levels were positively associated with insulin resistance, making it a key predictor of gestational diabetes [56, 57]. IDO is the rate-limiting enzyme in tryptophan metabolism, and responsible for generating kynurenine. Similarly, the kynurenine to tryptophan ratio (K/T) can reflect indoleamine-2,3-dioxygenase 1 activity [54]. Inhibiting IDO expression can also effectively improve insulin resistance [55, 58]. In our study, we observed relatively lower serum kynurenine, IDO, and K/T levels in LGG-fed sows. Interestingly, a positive correlation was identified between kynurenine and fasting plasma glucose levels. This suggested that increased kynurenine may be one cause of insulin resistance in sows during late pregnancy. A previous mouse study reported a significant increase in maternal serum kynurenine levels and insulin resistance in late pregnancy when compared to d 0 of gestation, which was not alleviated until the end of lactation [55]. Similar results were identified in our study, where serum kynurenine levels in sows increased continuously from late gestation to lactation. Additionally, our findings agreed with findings in mice and children with type 1 diabetes, indicating that regardless of increased or decreased serum kynurenine levels, it appears to be stable in the levels of tryptophan [55, 59]. This observation may be related to changes in other tryptophan metabolic pathways, because kynurenine, indole, and serotonin are all tryptophan metabolites, and when kynurenine is decreased or increased, this may cause fluctuations in other metabolic pathways. Correlation analysis revealed a notable negative relationship between *Lactobacillus* abundance and kynurenine levels, consistent with observations in a pregnant mouse model [55]. Mechanistically, reactive oxygen species produced by *Lactobacillus* may inhibit IDO synthesis, which in turn inhibits kynurenine production [60, 61]. We observed that alleviated insulin resistance in LGG-fed sows during late pregnancy and lactation was linked to reduced BCAA and kynurenine production.

The maternal gut microbiota has a long-term impact on establishing the gut microbiota in offspring [12, 62]. Beneficial gut bacteria are key to maintaining intestinal health and systemic immunity [63]. As expected, we showed that maternal LGG supplementation significantly increased the abundance of the beneficial bacteria *NK4A214_group* and decreased the relative abundance of the harmful bacteria *Streptococcus* and *Klebsiella* in piglets at d 10 of lactation. However, no significant intestinal flora differences were observed between groups on d 21 of lactation. This could be attributed to the dynamic nature of gut microbiota establishment in newborn piglets, which is influenced by various factors [64]. Critically, a stable intestinal microflora requires more complex flora characteristics to resist and adapt to external environmental changes [17]. Gut microbial co-occurrence network data based on core genera in LGG piglets were more complex than CON piglets on d 10 of lactation. This observation suggested that LGG supplementation in sows exerted positive roles on gut microbiota establishment in early-offspring piglets. Piglet viability in early life is influenced by bioactive factors found in umbilical cord blood and breast milk. Specifically, viability is subject to maternally derived immuno-active substances, such as IgG and IgM [65]. We observed that LGG significantly increased IgG and IgM levels in cord blood but not in milk. Similarly, a previous clinical study reported that LGG supplementation in pregnancy potentially influenced fetal immune parameters and immunomodulatory factors in breast milk [66]. Additionally, serum inflammatory cytokine changes in LGG piglets suggested potential health benefits from maternal LGG supplementation.

## Conclusions

In our study, LGG critically mitigated insulin resistance in gestating and lactating sows, with a significant finding showing that LGG affected BCAA or kynurenine metabolism by regulating specific microorganisms (e.g., *Bacteroides* and *Lactobacillus*) and ultimately affecting insulin resistance and lactation performance in sows. Increased insulin sensitivity stimulated heightened feed intake and lactose synthesis, ultimately fostering increased milk production and heightening early survival rates in newborn piglets during lactation. Moreover, maternal LGG supplementation enhanced immunoglobulin levels in umbilical cord blood, providing robust support for improved neonatal piglet viability. Simultaneously, LGG-induced maternal effects favored healthy gut microbiota establishment in neonatal piglets, diminishing pathogenic microbes and inflammation during lactation. Our study has substantial academic and practical significance, providing valuable scientific insights and practical implications for the academic community and the swine industry. Additionally, the molecular mechanisms underpinning the interactions between gut microbes and amino acid metabolism, in improving insulin sensitivity, warrant further investigation.

### Supplementary Information

**Additional file 1:** **Table S1** Body weight and backfat of sows at day 60 of gestation; **Table S2** Effect of *Lactobacillus rhamnosus* GG supplementation on fecal short-chain fatty acids in sows during late gestation; **Table S3** Effect of *Lactobacillus rhamnosus* GG supplementation in sows on the gut microbiota network index of offspring piglets; **Fig. S1** Effect of *Lactobacillus rhamnosus* GG supplementation on the performance of sows; **Fig. S2** Volcano plot and PLS-DA under positive or negative ion modes; **Fig. S3** Effect of LGG supplementation on serum Kynurenine and IDO content in sows; **Fig. S4** LGG supplementation in sows influenced gut microbial community of offspring piglets; **Fig. S5** Effect of maternal LGG supplementation on milk immunoglobulin.

## Data Availability

All data of this study are included in this published article.

## Abbreviations

BCAA: Branched-chain amino acids
GLUT8: Glucose transporters 8
IDO: Indoleamine 2,3-dioxygenase
IL-10: Interleukin-10
IL-6: Interleukin-6
IgG: Immunoglobulin G
IgM: Immunoglobulin M
LGG: *Lactobacillus rhamnosus* GG
PMEC: Pig mammary epithelial cells
PcoA: Principal coordinate analysis
SCC: Somatic cell counts
SCFA: Short chain fat acids
TNF-α: Tumor necrosis factor-α
VIP: Variable important in projection
