# Supplementary material for: Probiotic Lactobacillus rhamnosus GG improves insulin sensitivity and offspring survival via modulation of gut microbiota and serum metabolite in a sow model
Source: J Anim Sci Biotechnol. 2024 Jul 2;15:89. doi: 10.1186/s40104-024-01046-z (PMC11218078; doi:10.1186/s40104-024-01046-z)
Supplement: Supplementary file 1 — Additional file 1: Table S1 Body weight and backfat of sows at day 60 of gestation; Table S2 Effect of Lactobacillus rhamnosus GG supplementation on fecal short-chain fatty acids in sows during late gestation; Table S3 Effect of Lactobacillus rhamnosus GG supplementation in sows on the gut microbiota network index of offspring piglets; Fig. S1 Effect of Lactobacillus rhamnosus GG supplementation on the performance of sows; Fig. S2 Volcano plot and PLS-DA under positive or negative ion modes; Fig. S3 Effect of LGG supplementation on serum Kynurenine and IDO content in sows; Fig. S4 LGG supplementation in sows influenced gut microbial community of offspring piglets; Fig. S5 Effect of maternal LGG supplementation on milk immunoglobulin. [file 40104_2024_1046_MOESM1_ESM.docx]

Additional file 1

Table S1 Body weight and backfat of sows at day 60 of gestation

| Item | Treatments | | *P*-value |
| --- | --- | --- | --- |
|  | CON | LGG |  |
| *n* | 10 | 10 |  |
| Body weight, kg | 226.43 ± 16.00 | 225.67 ± 18.63 | 0.92 |
| Back fat, mm | 16.40 ± 2.82 | 16.85 ± 3.53 | 0.76 |

Data are expressed as mean with standard error of mean (SEM). The student’s *t*-test was applied to compare the difference between the two groups. Significant difference was declared at *P* < 0.05

Table S2 Effect of Lactobacillus rhamnosus GG supplementation on fecal short-chain fatty acids in sows during late gestation

| SCFA, μmol/g | Treatments | | *P*-value |
| --- | --- | --- | --- |
|  | CON | LGG |  |
| Acetate | 33.80 ± 5.40 | 29.10 ± 4.20 | 0.17 |
| Propionate | 14.70 ± 2.30 | 13.90 ± 0.30 | 0.54 |
| Butyrate | 8.00 ± 4.70 | 6.00 ± 2.00 | 0.27 |
| Isobutyrate | 1.60 ± 0.50 | 1.80 ± 0.10 | 0.15 |
| Valerate | 1.60 ± 0.30 | 2.90 ± 3.80 | 0.39 |
| Isovalerate | 3.20 ± 0.40 | 3.30 ± 0.40 | 0.40 |

Data are expressed as mean with standard error of mean (SEM). The student’s t-test was applied to compare the difference between the two groups. Significant difference was declared at *P* < 0.05

Table S3 Effect of *Lactobacillus rhamnosus* GG supplementation in sows on the gut microbiota network index of offspring piglets

| Items | Treatments | |
| --- | --- | --- |
|  | CON | LGG |
| GD | 0.037 | 0.040 |
| AD | 10.986 | 11.866 |
| APL | 2.810 | 3.437 |

GD, graph density; AD, average degree; APL, average path length

Fig. S1 Effect of Lactobacillus rhamnosus GG supplementation on the performance of sows. Body weight, body lipid and body protein loss of sows during lactation (A). Average daily feed intake and milk yield of sows during lactation (0–21 d). Litter weight gain (B) and survival rate of piglets (C). BW, body weight; BLL, body lipid loss; BPL, body protein loss. Piglet mortality was statistically analyzed using the Chi-square test. Data were presented as means ± SEM. * means *P* < 0.05, and ** means *P* < 0.01, ns means not significant (*P* > 0.05)

Fig. S2 Volcano plot and PLS-DA under positive or negative ion modes. Volcano plot under positive or negative ion modes (A). PLS-DA under positive or negative ion modes (B)

Fig. S3 Effect of LGG supplementation on serum Kynurenine and IDO content in sows. G90 means day 90 of gestation, L10 and L21 means day 10 and 21 of lactation. Data were presented as means ± SEM. * means *P* < 0.05, and ** means *P* < 0.01, ns means not significant (*P* > 0.05)

Fig. S4 LGG supplementation in sows influenced gut microbial community of offspring piglets. Alpha diversity of the gut microbiota including observed species, ACE and Chao 1 index (A). Relative abundance of significantly different genera on day 10 of lactation (B). Wilcoxon test was used to evaluate statistical differences. * means *P* < 0.05, and ** means *P* < 0.01. ns means not significant (*P* >0.05)

Fig. S5 Effect of maternal LGG supplementation on milk immunoglobulin. * Means *P* < 0.05, ** means *P* < 0.01 and ns means not significant (*P* > 0.05)
